# Urbanization creates diverse aquatic habitats for immature mosquitoes in urban areas

**DOI:** 10.1038/s41598-019-51787-5

**Published:** 2019-10-25

**Authors:** André B. B. Wilke, Catherine Chase, Chalmers Vasquez, Augusto Carvajal, Johana Medina, William D. Petrie, John C. Beier

**Affiliations:** 1 Department of Public Health Sciences, Miller School of Medicine, University of Miami, Miami, FL USA; 2 Miami-Dade County Mosquito Control Division, Miami, FL United States of America

**Keywords:** Biodiversity, Community ecology, Ecological epidemiology, Ecosystem ecology, Urban ecology

## Abstract

Global increases in temperatures and urbanization are impacting the epidemiology of mosquito-borne diseases. Urbanization processes create suitable habitats for vector mosquitoes in which there are a reduced number of predators, and human hosts are widely available. We hypothesize that mosquito vector species, especially *Aedes aegypti*, are locally concentrated primarily in those specific habitats at the neighborhood levels that provide suitable conditions and environmental resources needed for mosquito survival. Determining how mosquito vector species composition and abundance depend on environmental resources across habitats addresses where different types of vector control need to be applied. Therefore, our goal was to analyze and identify the most productive aquatic habitats for mosquitoes in Miami-Dade County, Florida. Immature mosquito surveys were conducted throughout Miami-Dade County from April 2018 to June 2019, totaling 2,488 inspections. Mosquitoes were collected in 76 different types of aquatic habitats scattered throughout 141 neighborhoods located in the urbanized areas of Miami-Dade County. A total of 44,599 immature mosquitoes were collected and *Ae. aegypti* was the most common and abundant species, comprising 43% of all specimens collected. *Aedes aegypti* was primarily found in buckets, bromeliads, and flower pots, concentrated in specific neighborhoods. Our results showed that aquatic habitats created by anthropogenic land-use modifications (e.g., ornamental bromeliads, buckets, etc.) were positively correlated with the abundance of *Ae. aegypti*. This study serves to identify how vector mosquitoes utilize the resources available in urban environments and to determine the exact role of these specific urban features in supporting populations of vector mosquito species. Ultimately, the identification of modifiable urban features will allow the development of targeted mosquito control strategies optimized to preventatively control vector mosquitoes in urban areas.

## Introduction

Global increases in temperatures and urbanization are impacting the epidemiology of mosquito-borne diseases^1^, resulting in severe outbreaks, even in formerly non-endemic areas^2–5^. Urbanization consists of altering the natural environment to make it more suitable for human populations and to accommodate both the growth of the local population and people moving from rural areas to cities^6,7^. Importantly, urbanization processes create suitable habitats for vector mosquitoes in which there are a reduced number of predators, and human hosts are wide available^6–9^. Public health efforts to control mosquito-borne diseases rely on mosquito control, which can achieve local success but generally is not enough to prevent arbovirus outbreaks.

Miami-Dade County, Florida is at risk for several arbovirus outbreaks including dengue (DENV), West Nile (WNV), chikungunya (CHIKV), Zika (ZIKV), and yellow fever (YFV) viruses that have occurred in past decades^10–15^. During the 2016 ZIKV outbreak, where there were locally acquired cases^16^; the virus was introduced to Miami on multiple occasions in different areas^17^.

Miami has complex environmental and socioeconomic features. Miami is one of the most important gateways to the U.S. due to an increased flow of people coming and going from endemic areas in the Caribbean region and Latin America, substantially increasing the risk of arbovirus introduction. In addition, Miami has the appropriate conditions for mosquitoes year-round, as the tropical monsoon climate is highly conducive for mosquitoes even during the winter^18^. Miami is also undergoing intense increases in urbanization^19,20^ that is impacting the population dynamics of vector mosquitoes and subsequently the risk of arbovirus transmission^21,22^.

Recent findings exposed the unexpected scenario that *Aedes* (*Stegomyia*) *aegypti* (Linnaeus, 1762) are successfully using ornamental bromeliads as larval habitats in Miami-Dade County, Florida^21^. Furthermore, subsequent studies on construction sites and tire shops in urban areas of Miami-Dade County showed that vector mosquitoes are breeding in high numbers in these areas. Results also showed reduced biodiversity of species in these habitats sheltering almost exclusively *Ae. aegypti* and *Culex* (*Culex*) *quinquefasciatus* (Say, 1823)^20,23^. These findings highlight the need to determine how the abundance of immature populations of vector mosquito species at point source locations is related to both features of the local environment and availability of breeding sites, representing vital resources needed by mosquito species for them to exist and propagate in definable urban habitats.

We hypothesize that mosquito vector species, especially *Ae. aegypti*, are locally concentrated primarily in those specific habitats at the neighborhood levels that provide suitable conditions and environmental resources needed for mosquito survival. Determining how mosquito vector species composition and abundance depend on environmental resources across habitats addresses where different types of vector control need to be applied. Therefore, our goal was to analyze and identify the most productive aquatic habitats for mosquitoes in Miami-Dade County, Florida.

## Results

**Figure 1:**
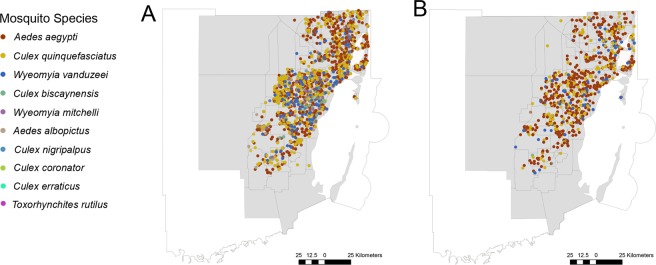
Map displaying the distribution of immature mosquitoes collected in Miami-Dade County, Florida for (**A**) larvae and (**B**) Pupae. Each color represents a mosquito species. Urban areas are displayed in gray. The figure was produced using ArcGIS 10.2 (Esri, Redlands, CA), using freely available layers from the Miami-Dade County’s Open Data Hub— https://gis-mdc.opendata.arcgis.com/.

Mosquitoes were collected in 76 different types of aquatic habitats (Supplementary Table 1) scattered throughout 141 neighborhoods located in the urbanized areas of Miami-Dade County. A total of 44,599 immature mosquitoes were collected, from which 19,206 were *Ae. aegypti* larvae and 2,997 pupae, 325 *Aedes* (*Stegomyia*) *albopictus* (Skuse, 1895) larvae and 65 pupae, 1.736 *Culex* (*Micraedes*) *biscaynensis* (Zavortink & O’Meara, 1999) larvae and 19 pupae, 212 *Culex* (*Culex*) *coronator* (Dyar & Knab, 1906) larvae and 4 pupae, 13 *Culex* (*Melanoconion*) *erraticus* (Dyar & Knab, 1906) larvae, 14,358 *Cx. quinquefasciatus* larvae and 1,193 pupae, 174 *Culex* (*Culex*) *nigripalpus* (Theobald, 1901) larvae and 3 pupae, 873 *Wyeomyia* (*Wyeomyia*) *mitchelli* (Theobald, 1905) larvae and 129 pupae, 3,054 *Wyeomyia* (*Wyeomyia*) *vanduzeei* (Dyar & Knab, 1906) larvae and 236 pupae, and 2 *Toxorhynchites* (*Lynchiella*) *rutilus* (Dyar and Knab, 1869) larvae (Fig. 1, Table 1, Supplementary Fig. S1).

**Table 1:** Immature mosquito species collected in Miami-Dade County from April 2018 to June 2019.

Neighborhood	Number of Inspections	*Aedes aegypti*		*Aedes albopictus*		*Culex biscaynensis*		*Culex coronator*		*Culex erraticus*		*Culex quinquefasciatus*		*Culex nigripalpus*		*Wyeomyia mitchelli*		*Wyeomyia vanduzeei*		*Toxorhynchites rutilus*	
		L	P	L	P	L	P	L	P	L	P	L	P	L	P	L	P	L	P	L	P
Auburdale	6	26	0	0	0	0	0	0	0	0	0	109	0	0	0	0	0	6	3	0	0
Aventura	7	16	9	0	0	0	0	6	0	0	0	118	0	0	0	0	2	0	0	0	0
Bal Harbor	2	8	0	0	0	0	0	0	0	0	0	4	8	0	0	0	0	0	0	0	0
Bay Harbor Island	6	50	22	0	0	0	0	0	0	0	0	3	46	1	0	0	0	0	0	0	0
Bay Shore	5	15	26	0	0	0	0	0	0	0	0	0	0	0	0	0	0	0	1	0	0
Bay Village	2	50	0	1	0	0	0	0	0	0	0	44	0	0	0	0	0	0	0	0	0
Bird Drive Basin	59	178	25	0	0	3	0	0	0	0	0	910	15	4	0	0	0	30	3	0	0
Biscayne Park	11	84	17	0	0	0	0	3	0	0	0	46	0	0	0	0	0	80	0	0	0
Biscayne Point	4	11	2	0	0	0	0	0	0	0	0	2	4	0	0	0	0	0	0	0	0
Blue Lagoon	5	21	2	0	0	0	0	0	0	0	0	17	3	0	0	0	0	0	0	0	0
Brickell	5	12	23	0	0	0	0	0	0	0	0	17	9	0	0	0	0	0	0	0	0
Brownsville	24	157	49	4	0	0	0	0	0	0	0	130	38	1	0	0	0	0	7	0	0
Buena Vista	23	264	39	0	0	0	0	0	0	0	0	309	18	0	0	0	0	28	1	0	0
Bunche Park	5	99	1	0	0	0	0	0	0	0	0	0	0	0	0	0	0	0	1	0	0
C-9 Basin Area	1	18	0	0	0	0	0	0	0	0	0	0	3	0	0	0	0	0	0	0	0
Calusa	25	76	34	0	0	19	0	2	0	0	0	316	6	0	0	0	0	82	2	0	0
Carol City	43	373	56	4	0	1	0	0	0	0	0	380	2	0	0	7	2	14	2	0	0
Catalina Lakes	21	136	18	0	0	3	0	0	0	0	0	24	19	0	0	22	0	15	1	0	0
Central Downtown	6	10	4	0	0	0	0	1	0	0	0	13	4	0	0	1	0	19	0	0	0
Central Gables	6	5	5	0	0	0	0	0	0	0	0	8	0	0	0	0	0	29	0	0	0
Civic Center	33	192	25	2	0	0	0	1	0	13	0	322	46	0	0	0	1	13	0	0	0
Coastal Wetland	2	1	0	0	0	0	0	0	0	0	0	3	0	0	0	0	0	0	0	0	0
Coral Terrace North	8	17	11	0	0	0	0	0	0	0	0	329	2	0	0	0	0	1	0	0	0
Coral Terrace South	23	200	16	0	0	0	0	0	0	0	0	78	0	0	0	4	35	8	1	0	0
Country Club Of Miami	7	30	8	0	0	0	0	0	0	0	0	37	1	0	0	0	0	0	0	0	0
Cutler	88	331	80	5	0	209	0	0	1	0	0	229	63	0	0	9	0	271	5	0	0
Cutler Ridge	22	1386	25	0	1	0	0	0	0	0	0	60	0	0	0	0	0	4	0	0	0
Dadeland	9	208	22	0	0	14	0	0	0	0	0	43	0	0	0	1	0	9	0	0	0
Doral Area	11	80	28	0	0	1	0	0	0	0	0	53	1	0	0	0	0	3	0	0	0
Douglas Park	6	28	4	0	0	0	0	0	0	0	0	1	0	0	0	0	0	0	2	0	0
East Goulds	44	321	18	0	0	0	0	0	0	0	0	234	25	0	0	1	0	23	12	0	0
East Homestead	6	15	6	0	0	0	5	0	0	0	0	12	0	0	0	0	1	0	0	0	0
East Kendall	59	334	52	3	1	230	1	1	0	0	0	134	36	5	0	104	1	108	6	0	0
East Liberty City	23	323	13	6	0	0	0	0	0	0	0	132	0	6	0	0	0	0	0	0	0
East Naranja	13	187	10	27	0	0	0	0	0	0	0	80	2	25	0	0	0	0	0	0	0
East South Miami	4	121	20	0	0	0	0	0	0	0	0	0	0	0	0	0	2	23	0	0	0
East South Miami City	4	4	27	0	0	0	0	0	0	0	0	33	0	0	0	0	0	6	0	0	0
East Turnpike Area	2	3	1	0	0	0	0	0	0	0	0	4	0	0	0	0	0	49	0	0	0
Eastern Shores	20	168	8	0	0	0	0	0	0	0	0	8	3	0	0	0	0	2	2	0	0
El Portal	4	8	0	0	0	0	0	0	0	0	0	9	0	0	0	6	0	23	10	0	0
Flagler Westside	16	92	33	0	0	0	0	0	0	0	0	42	5	9	0	7	0	3	0	0	0
Flamingo	7	5	22	0	0	0	0	0	0	0	0	14	17	0	0	0	0	62	0	0	0
Florida City	10	111	8	1	0	0	0	0	0	0	0	34	0	6	0	0	0	0	4	0	0
Gables Bayfront	12	54	34	0	0	65	0	0	0	0	0	13	0	0	0	0	0	14	11	0	0
Golden Glades	27	45	41	109	0	0	0	50	0	0	0	94	3	0	0	125	0	53	1	0	0
Granada	12	266	8	0	0	0	0	0	0	0	0	134	8	0	0	4	0	0	0	0	0
Grapeland	10	255	3	0	0	0	0	0	0	0	0	4	4	0	0	0	0	0	0	0	0
Hammocks	56	231	70	0	1	1	0	0	0	0	0	294	11	32	0	0	0	24	3	0	0
Hialeah - Area 1	5	31	6	0	0	0	0	0	0	0	0	75	0	0	0	0	0	0	0	0	0
Hialeah - Area 2	11	31	15	0	0	0	0	0	0	0	0	1	0	0	0	0	0	28	0	0	0
Hialeah - Area 3	7	33	1	0	0	0	0	0	0	0	0	33	17	0	0	1	0	5	0	0	0
Hialeah - Area 4	3	18	1	0	0	0	0	0	0	0	0	0	0	0	0	0	0	9	0	0	0
Hialeah - Area 5	1	1	1	0	0	0	0	0	0	0	0	2	3	0	0	0	0	0	0	0	0
Hialeah - Area 6	2	1	0	0	0	0	0	0	0	0	0	6	0	0	0	0	1	0	0	0	0
Hialeah - Area 7	13	79	7	2	0	0	0	0	0	0	0	151	2	2	0	0	0	0	0	0	0
Hialeah Gardens	5	36	4	0	0	0	0	0	0	0	0	0	0	0	0	0	0	0	14	0	0
Homestead	18	56	29	0	0	2	1	0	0	0	0	327	3	0	0	0	2	30	0	0	0
Homestead Base	2	0	0	0	0	0	0	0	0	0	0	22	1	0	0	0	0	0	0	0	0
Homestead Lakes	7	21	2	0	0	0	0	0	0	0	0	31	1	0	0	0	1	0	0	0	0
Horse Country	7	7	3	0	0	0	0	0	0	0	0	32	0	0	0	0	0	2	0	0	0
Interama	1	11	3	0	0	0	0	0	0	0	0	0	0	0	0	0	0	0	0	0	0
Ives Estate	15	175	16	0	0	0	0	3	0	0	0	168	6	0	0	0	0	0	0	0	0
Kendale Lakes	71	430	103	0	1	34	0	0	0	0	0	289	54	0	0	10	0	20	4	0	0
Kendall	120	804	160	8	0	438	0	0	0	0	0	309	23	4	0	87	0	201	9	0	0
Kendall North	15	36	26	0	0	0	0	0	0	0	0	108	0	0	0	0	2	12	1	0	0
Key Biscayne - Bay Area	11	148	9	0	0	0	0	0	0	0	0	10	0	4	0	0	0	0	5	0	0
Keystone Islands	15	122	22	2	0	0	0	0	0	0	0	60	0	21	0	28	0	1	5	0	0
La Gorce	3	152	3	0	0	0	0	0	0	0	0	0	2	0	0	0	0	0	0	0	0
Leisure City Area	34	239	14	1	0	25	1	0	0	0	0	85	3	0	0	11	0	74	0	0	0
Little Havana	11	67	0	0	0	0	0	2	0	0	0	340	0	0	0	2	0	10	0	0	0
Little River	9	40	0	0	0	0	0	0	0	0	0	115	1	1	0	0	0	0	1	0	0
Management Area - 1	9	87	5	2	0	0	0	42	0	0	0	315	3	0	0	0	0	0	0	0	0
Marbella Park	7	34	20	0	0	0	0	0	0	0	0	93	0	0	0	0	0	0	0	0	0
Metro-Lindgren	33	205	51	0	0	3	0	16	0	0	0	232	22	0	0	28	0	21	2	0	0
Miami Industrial	7	22	17	0	0	0	0	0	0	0	0	1	0	0	0	0	0	6	0	0	0
Miami Lakes	18	176	16	0	0	0	0	0	2	0	0	103	11	0	0	0	0	4	12	0	0
Miami Shores	10	87	12	0	25	0	0	0	0	0	0	20	17	0	0	0	0	6	0	0	0
Miami Springs - Area 1	16	22	9	0	0	0	0	0	0	0	0	66	47	0	0	3	0	20	2	0	0
Miami Springs - Area 2	26	93	36	0	0	11	0	0	0	0	0	29	6	7	0	14	0	3	2	0	0
Miami Springs - Area 3	25	168	53	3	2	0	0	0	0	0	0	24	2	0	0	2	0	36	1	0	0
Naranja	15	82	4	0	0	0	0	0	0	0	0	39	8	0	0	0	3	3	0	0	0
Nautilus	3	59	11	0	0	0	0	0	0	0	0	0	0	0	0	0	0	0	2	0	0
Norland	18	266	27	0	0	3	0	30	0	0	0	32	0	0	0	3	0	6	0	0	0
Normandy Isle	29	275	8	0	0	0	1	0	0	0	0	119	1	0	0	0	0	0	2	0	0
North Bayfront	17	72	9	0	0	0	0	0	0	0	0	324	9	0	0	14	0	13	2	0	0
North Gables	8	50	1	0	0	0	6	0	0	0	0	4	26	0	0	7	0	0	4	0	0
North Grove	25	66	92	0	0	4	0	0	0	0	0	219	26	6	0	3	6	114	0	0	0
North Hialeah Gardens	5	30	3	0	0	0	0	0	0	0	0	2	0	0	0	0	0	0	0	0	0
North Opalocka	6	99	4	0	0	0	0	0	0	0	0	27	0	0	0	0	0	0	3	0	0
North Palm Springs	5	47	0	0	0	0	0	0	0	0	0	14	0	0	0	0	0	0	0	0	0
North Redlands	104	1103	118	35	0	120	0	25	1	0	0	818	52	9	0	50	42	25	20	2	0
North Shore	4	69	7	0	0	0	0	0	0	0	0	49	0	0	0	12	0	0	2	0	0
Oceanpoint	6	52	2	0	0	0	0	0	0	0	0	1	0	0	0	0	0	0	0	0	0
Ojus	24	77	6	0	0	0	0	0	0	0	0	145	0	0	0	0	13	69	0	0	0
Olympia Heights	6	35	6	1	0	0	0	0	0	0	0	0	0	0	0	0	0	1	0	0	0
Omni - Boulevard	5	43	0	0	0	0	0	0	0	0	0	12	0	0	0	0	0	12	0	0	0
Opalocka City	12	218	26	0	0	0	0	2	0	0	0	43	0	1	0	40	0	1	11	0	0
Overtown	3	4	6	0	0	0	0	0	0	0	0	12	0	0	0	0	0	13	0	0	0
Perrine	35	153	30	0	0	73	0	0	0	0	0	257	0	0	1	1	0	106	4	0	0
Richmond	23	292	47	0	3	38	0	2	0	0	0	131	4	0	0	2	1	76	0	0	0
Saga Bay	18	147	11	31	0	0	0	0	0	0	0	121	3	0	0	39	0	17	0	0	0
Scott Lake	16	120	111	16	0	0	0	0	0	0	0	204	0	0	0	0	0	0	0	0	0
Shenandoah	28	214	23	0	0	4	0	0	0	0	0	87	5	0	0	3	0	95	2	0	0
South Gables	5	20	5	0	0	0	0	0	0	0	0	6	0	0	0	0	3	0	0	0	0
South Golden Glades	18	122	42	0	0	0	0	3	0	0	0	76	4	0	0	0	0	37	1	0	0
South Grove	18	222	33	0	0	19	0	0	0	0	0	8	9	0	0	15	0	51	7	0	0
South Miami Heights	41	254	17	0	0	2	0	0	0	0	0	405	29	0	0	2	0	65	1	0	0
South Naranja	6	38	3	0	0	0	0	0	0	0	0	4	0	0	0	0	0	12	0	0	0
South North Miami Beach	19	90	19	0	0	0	0	7	0	0	0	217	38	0	0	0	0	143	0	0	0
Sunny Isles	5	13	0	0	0	0	0	0	0	0	0	52	8	0	0	0	0	0	0	0	0
Sunset East	15	191	37	6	0	12	0	0	0	0	0	146	4	0	0	62	0	0	0	0	0
Sunset Islands	4	3	1	0	0	0	0	0	0	0	0	8	0	0	0	0	0	0	2	0	0
Sunset West	42	469	25	8	5	99	0	0	0	0	0	129	38	3	0	11	2	101	4	0	0
Surfside	6	38	4	0	0	0	0	0	0	0	0	10	8	0	0	0	0	0	4	0	0
Sweetwater	8	48	3	0	0	0	0	0	0	0	0	71	10	0	0	0	0	0	2	0	0
Tamiami	44	259	42	4	0	0	0	0	0	0	0	234	14	0	0	11	0	36	3	0	0
Tamiami - Lindgren	41	225	20	0	0	0	0	0	0	0	0	211	45	0	0	0	0	17	2	0	0
Transitional Area	1	0	0	0	0	0	0	0	0	0	0	0	9	0	0	0	0	0	0	0	0
University	15	161	24	0	0	0	0	0	0	0	0	102	0	0	0	0	0	13	0	0	0
Venetian Islands	44	664	94	0	11	0	0	0	0	0	0	184	4	0	0	0	3	0	0	0	0
West Ave	2	0	5	0	0	0	0	0	0	0	0	10	0	0	0	0	0	8	0	0	0
West Cutler Area	13	17	14	0	0	18	0	0	0	0	0	149	15	0	0	0	0	16	0	0	0
West Flagler	20	179	78	2	11	0	0	0	0	0	0	82	35	0	0	0	0	22	0	0	0
West Goulds	4	19	12	0	0	0	0	0	0	0	0	1	7	0	0	0	0	14	0	0	0
West Homestead	11	83	13	10	0	0	0	0	0	0	0	138	4	0	0	0	0	0	1	0	0
West Kendall	19	38	7	0	0	0	3	0	0	0	0	58	9	0	0	8	0	85	0	0	0
West Lake Lucerne	5	23	21	0	0	0	0	0	0	0	0	0	21	0	0	0	4	1	0	0	0
West Little River	26	408	26	30	0	12	1	0	0	0	0	42	6	0	0	0	1	25	2	0	0
West Miami	4	72	8	0	0	0	0	0	0	0	0	2	3	0	0	1	0	0	0	0	0
West Miami Lakes	38	233	20	0	2	0	0	0	0	0	0	92	2	15	0	21	0	103	1	0	0
West Miami Shores	22	93	10	0	0	0	0	1	0	0	0	79	4	0	0	26	0	59	4	0	0
West North Miami	11	100	21	0	0	0	0	0	0	0	0	194	6	0	0	15	0	37	0	0	0
West Quail Roost	23	260	54	1	1	0	0	1	0	0	0	204	6	0	0	10	0	0	3	0	0
West South Miami	13	117	13	0	0	7	0	0	0	0	0	18	7	6	0	4	0	1	3	0	0
West South Miami City	13	27	1	0	1	97	0	0	0	0	0	60	2	0	0	13	0	43	1	0	0
West Sweetwater	22	57	5	0	0	25	0	0	0	0	0	68	21	0	0	0	0	21	1	0	0
West Tamiami	18	116	26	0	0	0	0	0	0	0	0	52	0	0	0	0	0	2	0	0	0
Westchester	68	365	135	0	0	130	0	1	0	0	0	153	15	0	1	19	0	48	0	0	0
Westview	14	63	7	1	0	0	0	13	0	0	0	221	2	3	0	0	1	0	4	0	0
Westwood Lakes	21	68	3	0	0	12	0	0	0	0	0	115	6	3	0	1	0	37	0	0	0
Wynwood	33	421	28	0	0	2	0	0	0	0	0	170	42	0	1	3	0	16	2	0	0
Total	2,488	19206	2997	325	65	1736	19	212	4	13	0	14358	1193	174	3	873	129	3054	236	2	0

L = larvae; P = pupae.

**Figure 2:**
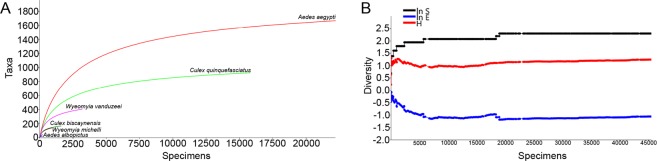
Biodiversity indices for the immature mosquitoes collected in Miami-Dade County, Florida from April 2018 to June 2019. (**A**) Individual rarefaction curves (Y-axis = number of species; X-axis = number of specimens); (**B**) Plots of cumulative SHE profiles (ln S, H and ln E). (Y-axis = diversity values for log abundance, Shannon index and log evenness; X-axis = number of specimens).

Based on the totality of collected mosquitoes, the individual rarefaction curves resulted in moderately high asymptotic curves for *Ae. aegypti* and *Cx. quinquefasciatus* with high degree of confidence for predicting the expected presence of those species for smaller samples. The cumulative SHE profiles indices reached stability after a short period of initial variation and yielded relatively low values for the Ln S, Ln E and H. These results are indicating an uneven mosquito assembly with low diversity and reduced richness of species in the urbanized areas of Miami (Fig. 2).

**Figure 3:**
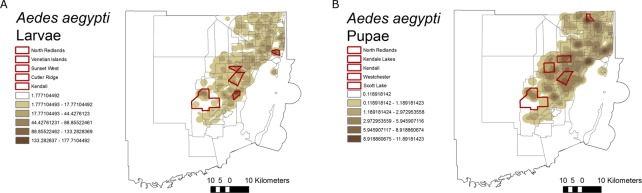
Heat map based on the relative abundance of *Aedes aegypti* larvae (**A**) and pupae (**B**) in Miami-Dade County, Florida. Highlighted in red are the neighborhoods with the highest abundance of *Ae. aegypti*. The figure was produced using ArcGIS 10.2 (Esri, Redlands, CA), using freely available layers from the Miami-Dade County’s Open Data Hub— https://gis-mdc.opendata.arcgis.com/.

*Aedes aegypti* was the most abundant and widespread mosquito species in Miami-Dade County. From the 141 neighborhoods surveyed in this study, *Ae. aegypti* larvae were found in 138 neighborhoods and pupae in 127 neighborhoods. However*, Ae. aegypti* were more concentrated in specific neighborhoods, and only in six were more than 500 specimens collected: Cutler Ridge, 1,386 larvae and 25 pupae; North Redlands, 1,103 larvae and 118 pupae; Kendall, 804 larvae and 160 pupae; Venetian Islands 664 larvae and 94 pupae; and Kendale Lakes, 430 larvae and 103 pupae (Fig. 3).

**Figure 4:**
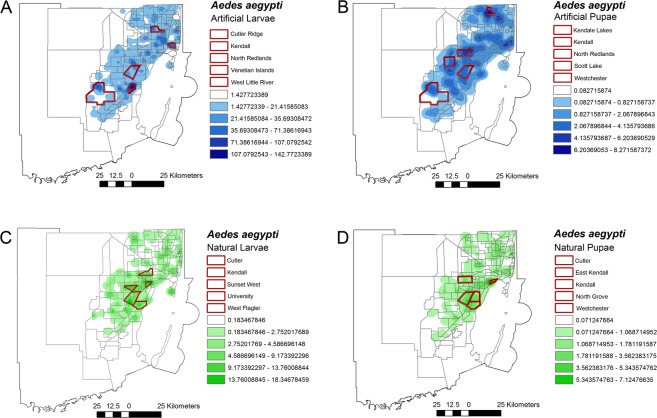
Heat map based on the relative abundance of *Aedes aegypti* breeding in natural and artificial habitats in Miami-Dade County, Florida. (**A**) Larvae and (**B**) pupae collected in artificial breeding habitats, and (**C**) Larvae and (**D**) pupae collected in natural breeding habitats. Highlighted in red are the neighborhoods with the highest abundance of *Ae. aegypti*. The figure was produced using ArcGIS 10.2 (Esri, Redlands, CA), using freely available layers from the Miami-Dade County’s Open Data Hub— https://gis-mdc.opendata.arcgis.com/.

Immature forms of *Ae. aegypti* were more abundantly found in artificial breeding sites than natural. A total of 15,701 larvae and 2,044 pupae were collected in artificial aquatic habitats while only 2,703 larvae and 850 pupae were collected in natural habitats. Interestingly, the most productive neighborhoods differed according to natural and artificial habitats, but in Kendall a high abundance of *Ae. aegypti* was shown in both natural and artificial habitats (Fig. 4).

The most productive aquatic habitats for *Ae. aegypti* in Miami-Dade County during this study were buckets, bromeliads, and flower pots, representing approximately 38% of all *Ae. aegypti* collected. The ten most productive breeding sites were responsible for approximately 67% of collected *Ae. aegypti* (Table 2).

**Table 2:** Most productive breeding sites for *Aedes aegypti* in Miami-Dade County, Florida.

Breeding Habitats	Larvae	Pupae	Total
Bucket	2,804	335	3139
Bromeliad	2,206	701	2907
Flower Pot	2,203	294	2497
Tire	1,901	165	2066
Fountain	1,050	141	1191
Plastic Container	1,015	77	1092
Storm Drain	401	273	674
Planter	508	74	582
Bird bath	353	48	401
Pot	298	60	358

**Figure 5:**
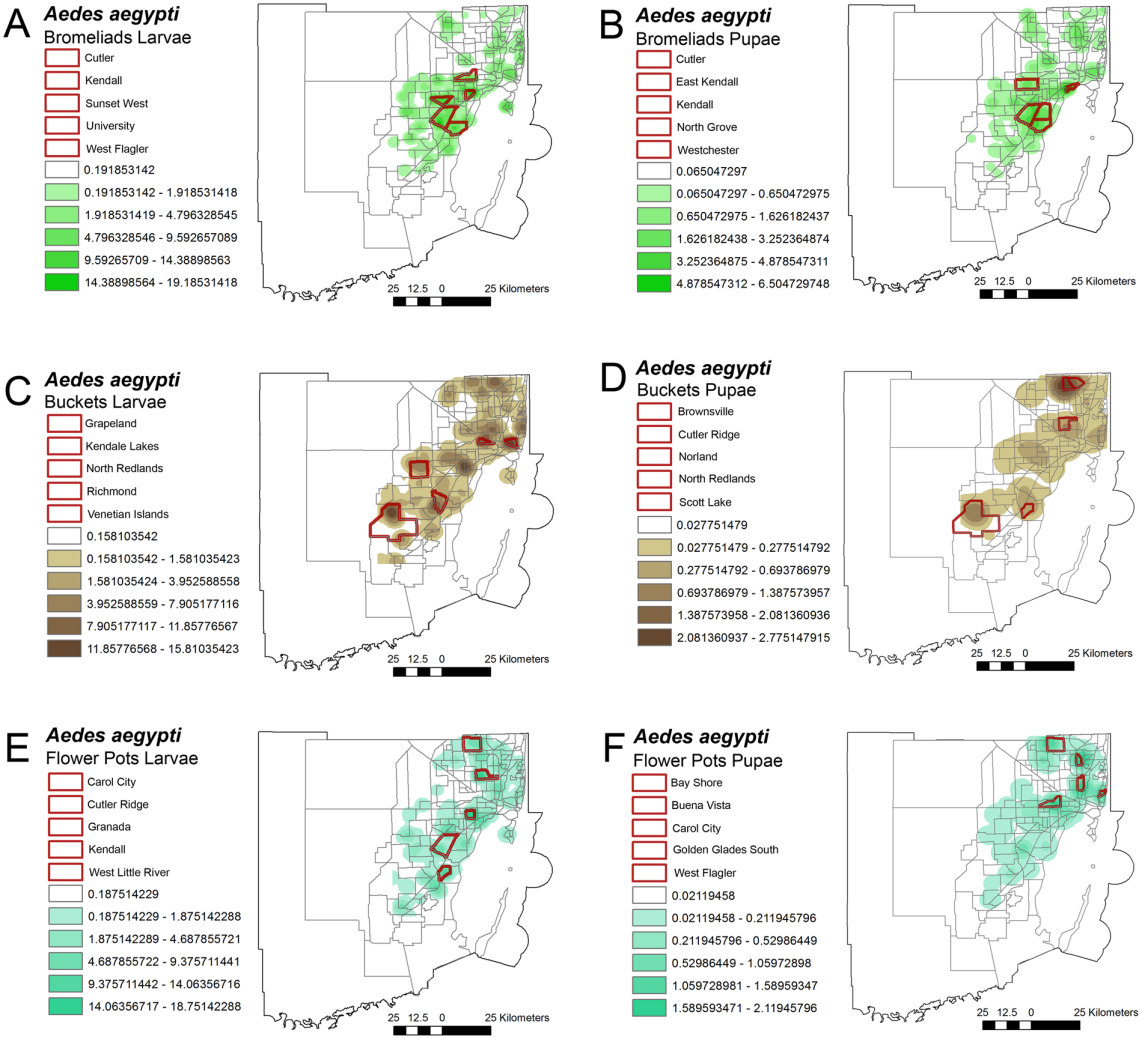
Most productive *Aedes aegypti* breeding habitats. (**A**) larvae and (**B**) pupae collected in bromeliads; (**C**) Larvae and (**D**) pupae collected in buckets and (**E**) larvae and (**F**) pupae collected in flower pots. The figure was produced using ArcGIS 10.2 (Esri, Redlands, CA), using freely available layers from the Miami-Dade County’s Open Data Hub— https://gis-mdc.opendata.arcgis.com/.

The three aquatic habitats in which *Ae. aegypti* was most abundantly found are common throughout Miami-Dade County. Bromeliads were responsible for supporting the development of *Ae. aegypti* in urban areas of Miami. These plants are common in highly urbanized areas and have been correlated with the production of *Ae. aegypti* larvae and pupae^21^. The relative abundance of larvae and pupae was moderately dissimilar regarding to point source location being more abundant in different neighborhoods (Fig. 5A,B).

The geospatial analysis revealed that buckets were found to be present in most neighborhoods directly overlapping with the relative abundance of immature *Ae. aegypti*. However, apart from the North Redlands the neighborhoods with higher number of larvae (Fig. 5C) were not the ones with the most pupae (Fig. 5D). Flower pots were the third most productive *Ae. aegypti* aquatic habitat, and apart from Kendall, were not correlated with the presence of bromeliads. Flower pots were found in highly urbanized areas such as Granada and as well as suburban areas such as Cutler Ridge (Fig. 5E,F).

## Discussion

Modifications of the natural environment alter the interactions between vector, host, and pathogen, which ultimately affects the epidemiology of vector-borne diseases^24^. These diseases are dependent on the natural environment, and environmental changes such as climate change, urbanization, and loss of biodiversity increase the risk of arbovirus transmission for the human population^6,18,25–29^. *Aedes aegypti* is the primary vector of DENV, ZIKV, and CHIKV and is well adapted to the urban environment of Miami-Dade, being present year-round^18^. Previous studies showed that this species is able to thrive in extreme urban environments such as construction sites and tire shops with limited sugar sources and few host species other than humans^20,23^. Our results show that mosquito vector species can be found in a wide range of aquatic habitats in the urban environments of Miami-Dade County. In these urban settings, practically any object that can hold water, from a deflated basketball to a Jet Ski or a storm drain, is a potential breeding site for vector mosquitoes.

Immature mosquitoes are widely distributed across Miami-Dade County and *Ae. aegypti* was by far the most common and abundant species, comprising 55.8% of all specimens collected during the timeframe of the study. The remaining seven species collected represent a much smaller proportion of the overall mosquito makeup of Miami-Dade County. Furthermore, larvae from the eight different species that were found, but only *Cx. quinquefasciatus* and *Ae. aegypti* were commonly found in the form of pupae, indicating that these species are more widespread in urban aquatic habitats than the remaining species found in this study.

No *Cx. nigripalpus* pupae were found in urban aquatic habitats indicating that this species may not be able to utilize these habitats successfully. Our findings are in agreement with previous findings in which immature *Cx. nigripalpus* specimens were not found breeding in aquatic habitats in urban environments in Miami^20,21,23^. Furthermore, adult *Cx. nigripalpus* are commonly collected in the edge of the incorporated areas of Miami but are rarely found in urban areas^18^. Therefore, immature *Cx. nigripalpus* collected in this study may be the result of specimens migrating from rural areas to urban areas but were unable to survive the harsh conditions of urban habitats.

*Aedes aegypti* was found in relatively high numbers throughout Miami-Dade County successfully breeding in aquatic habitats in diverse urban environments. However, it was primarily found in certain types of breeding habitats, responsible for supporting the development of *Ae. aegypti*, concentrated in the specific neighborhoods of Cutler Ridge, North Redlands, Kendall, Wynwood, and the Venetian Islands. The three most productive breeding sites for *Ae. aegypti*, in terms of numbers of immature mosquitoes produces, including buckets, bromeliads, and flower pots. Our results showed a clear correlation between the availability of breeding sites and the abundance of *Ae. aegypti* in these top five neighborhoods.

Not surprisingly, similar hotspots were discovered for both larvae and pupae, and these areas are where targeted mosquito control efforts should be most heavily implemented. Among these aquatic habitats responsible for driving the relative abundance of vector mosquitoes, special attention should be given to ornamental bromeliads. They have become an important breeding site for *Ae. aegypti* representing a challenge for vector mosquito control strategies in urban environments^21,30^.

*Aedes aegypti*’s opportunistic behavior allows it to utilize a wide range of breeding sites, both within the natural and artificial realm. It is clear from our larval surveillance data that more immatures were collected in artificial aquatic habitats than natural habitats, yet there are clear differences between the top five neighborhoods for natural and artificial habitats. For larvae found in artificial habitats, the highest densities of immature mosquitoes were found in North Redlands, Wynwood, Venetian Islands, Kendall, and Richmond Heights. However, larvae discovered in natural breeding sites were concentrated (albeit at lower abundances) in southeastern Miami-Dade County in the neighborhoods of Kendall, Cutler, Sunset West, University, and Richmond Heights.

Understanding the most productive breeding sites for *Ae. aegypti*, and other mosquito vector species, and where they are located within the county, is a powerful tool for targeted mosquito control. The number of immature mosquitoes produced per breeding site could be a useful tool in determining priorities in public health outreach and mosquito control efforts. It is far more desirable to control larvae than adults, and mosquito control practices should not solely prioritize adult control over larval control in order to achieve maximum effectiveness on mosquito control^31,32^.

However, it is important to understand that neighborhoods that produce mosquitoes from one specific breeding site may not produce many mosquitoes from other breeding sites, and human behavior is a large driver of this phenomenon. For example, it is evident that buckets played a strong role in immature *Ae. aegypti* abundance in the North Redlands, yet flower pots did not. North Redlands is an unincorporated agricultural area with a small human population, so it is logical that there is a high density of buckets contributing to the large abundance of immature *Ae. aegypti* mosquitoes, and very few flower pots being utilized as a breeding site.

In terms of bromeliads as a breeding site, it is evident that they play a crucial role in Kendall and the surrounding areas, possibly due to their ornamental nature in private gardens and the accompanying large human population in South Miami-Dade County. Accordingly, bromeliads do not contribute as strongly to North Redlands. This area’s small human population correlates to a lower density of bromeliads in the area, and therefore a minimal correlation between this breeding site and *Ae. aegypti* abundance. Understanding the connections between the locations of breeding sites in relation to human behavior is key to the development of more effective guided mosquito control strategies.

While *Ae. aegypti* is widespread throughout the county, its most productive breeding sites are modifiable and easily removed or avoided in urban environments. Buckets and containers can be dumped or turned over, and citizens can be educated on ornamental bromeliads as a potential breeding site. Education and outreach regarding these modifiable urban features could prove a valuable tool to control mosquito populations.

Due to the ability to thrive in urban areas, *Ae. aegypti* is increasing its presence and abundance worldwide^33^. The degradation of natural habitats positions the global human population at an overall increased risk for preventable outbreaks, particularly in urban areas, through increasingly severe outbreaks and the emergence of outbreaks in previously non-endemic areas^4,17,34^. Spread over an area of more than 6 thousand km^2^ and with more than 3 million residents, Miami-Dade is the most populous and third-largest county in Florida^35^. Miami’s large and ever-growing population, combined with its aforementioned proximity to endemic areas and appropriate climate for mosquito production year-round, positions the area in a unique situation for a high risk of vector-borne disease transmission and emergence^18^.

This study serves as a cornerstone for future studies that are needed to identify how vector mosquitoes utilize the resources available in urban environments and to determine the exact role of these specific urban features in supporting populations of vector mosquito species. Ultimately, the identification of modifiable urban features that will lead to the reduction of aquatic habitats for vector mosquitoes will allow the development of targeted mosquito control strategies optimized to preventatively control vector mosquitoes in urban areas.

## Methods

### Study area

Immature mosquito surveys were conducted in Miami-Dade County, Florida from April 2018 to June 2019, totaling 2,488 inspections. Surveys were requested by citizen complaints through 311 calls, automatically triggering the dispatch of a Mosquito Control inspector to actively search for potential mosquito aquatic habitats within a 50-meter radius from the original point-source location (Fig. 1, Supplementary Fig. S2). The 311 calls represent specific locations where residents deemed they had a serious mosquito problem and needed assistance from the County. Such 311 calls are normal for the State of Florida counties, but the information from site inspections is generally not used to direct mosquito control activities^36^. In this study, we had inspectors do larval searches from observed breeding habitats.

### Collection methods

Immature mosquitoes were collected by inspectors with the aid of manual plastic pumps (turkey basters) and entomological dippers, then stored for transport in plastic containers (100 ml) according to the breeding site where they were collected. All collected immature mosquitoes were transported to the Miami-Dade County Mosquito Control Laboratory. Mosquitoes were identified to species using taxonomic keys based on morphological characters^37^. Larvae were identified immediately after collection and all pupae were allowed to emerge as adults and then identified.

Since this study posed less than minimal risk to participants and did not involve endangered or protected species the Institutional Review Board at the University of Miami determined that the study was exempt from institutional review board assessment (IRB Protocol Number: 20161212).

### Breeding site categorization

Breeding sites were organized into two categories: (i) Category 1 - specific breeding habitat in which the specimens were collected; and (ii) Category 2 - artificial or natural to distinguish between man-made and natural features (Supplementary Table 2)^38,39^.

### Analysis

Biodiversity analyses were performed for all collected mosquitoes using individual rarefaction curves to compare mosquito diversity in samples with different sizes. The individual rarefaction curves were also used to provide an estimation of the number of species in samples with fewer specimens and to evaluate sampling sufficiency. Plots of cumulative profiles of species log abundance (ln S), Shannon index (H) and log evenness (ln E) (SHE) were also calculated for all samples. Samples were successively added to the model in chronologic order to assess variations in mosquito community and composition of species^40^. Analyses were carried out with 10,000 randomizations without replacement and a 95% confidence interval using Past software (v.3.16)^41,42^.

**Figure 6:**
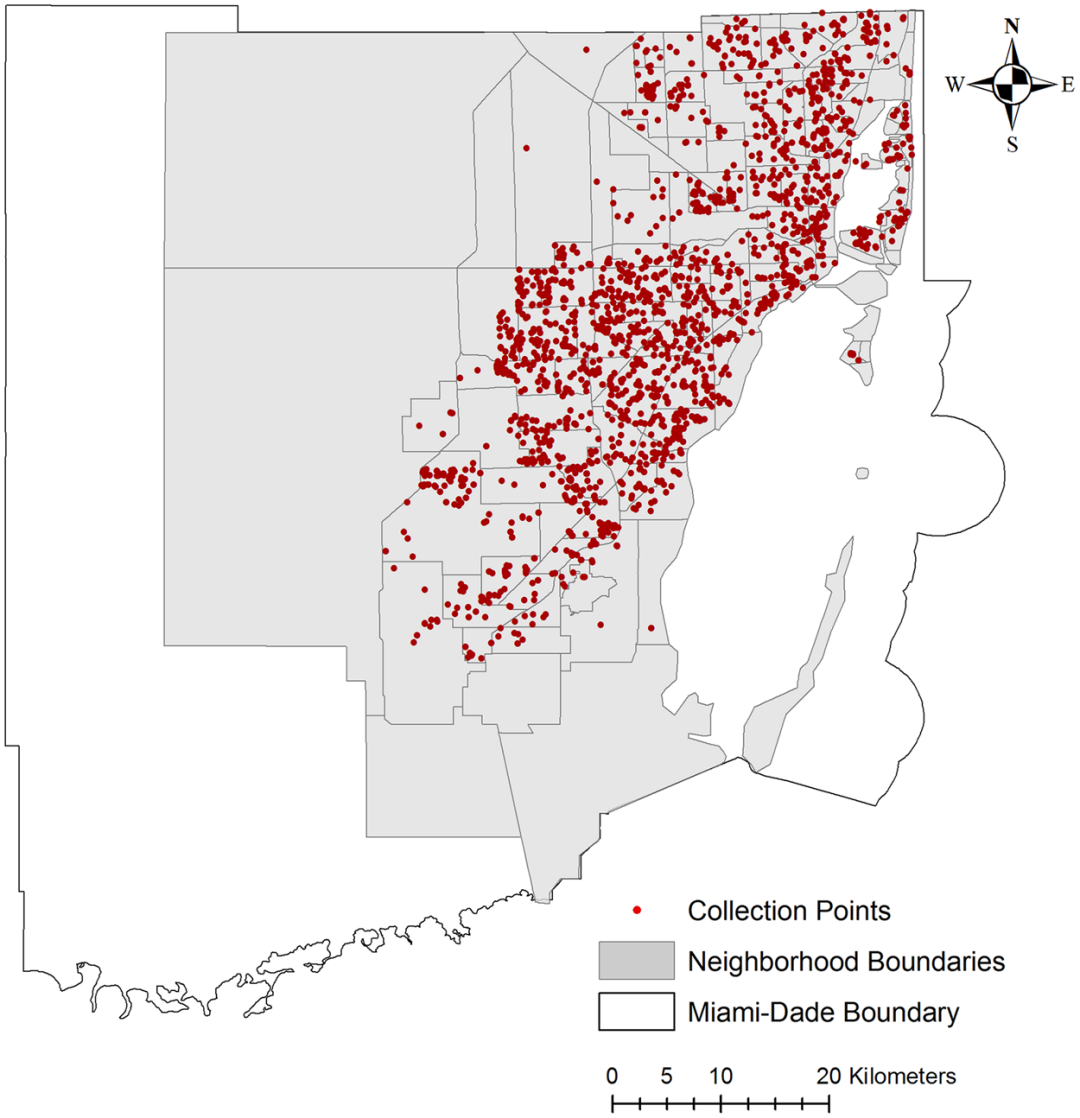
Map displaying immature mosquito collection points in Miami-Dade County, Florida. Neighborhoods are displayed in gray and collection points in red. The figure was produced using ArcGIS 10.2 (Esri, Redlands, CA), using freely available layers from the Miami-Dade County’s Open Data Hub— https://gis-mdc.opendata.arcgis.com/.

Figures 1, 3–6 were produced using ArcGIS (v.10.2) using maps freely available at www.census.gov and https://gis-mdc.opendata.arcgis.com/. Addresses of breeding sites from survey data were geocoded to map coordinates for consistency and confidentiality.

## Supplementary information

Supplementary Information

Supplementary Information

Supplementary Information

Supplementary Information
