# Supplementary figures and images for: Urbanization creates diverse aquatic habitats for immature mosquitoes in urban areas
Source: Sci Rep. 2019 Oct 25;9:15335. doi: 10.1038/s41598-019-51787-5 (PMC6814835; doi:10.1038/s41598-019-51787-5)

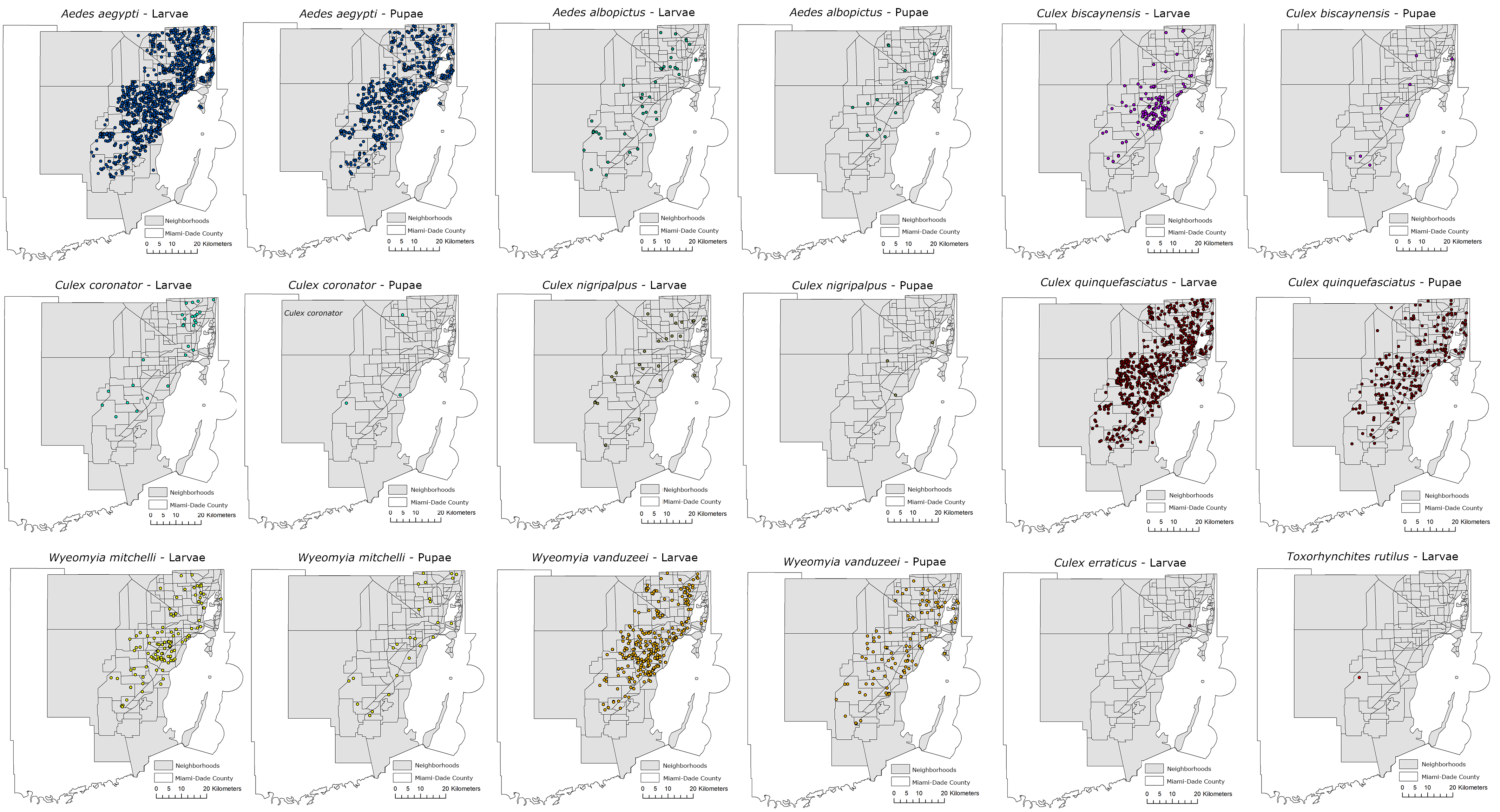

Supplement: Supplementary file 1 — Supplementary Information [file 41598_2019_51787_MOESM1_ESM.tif]

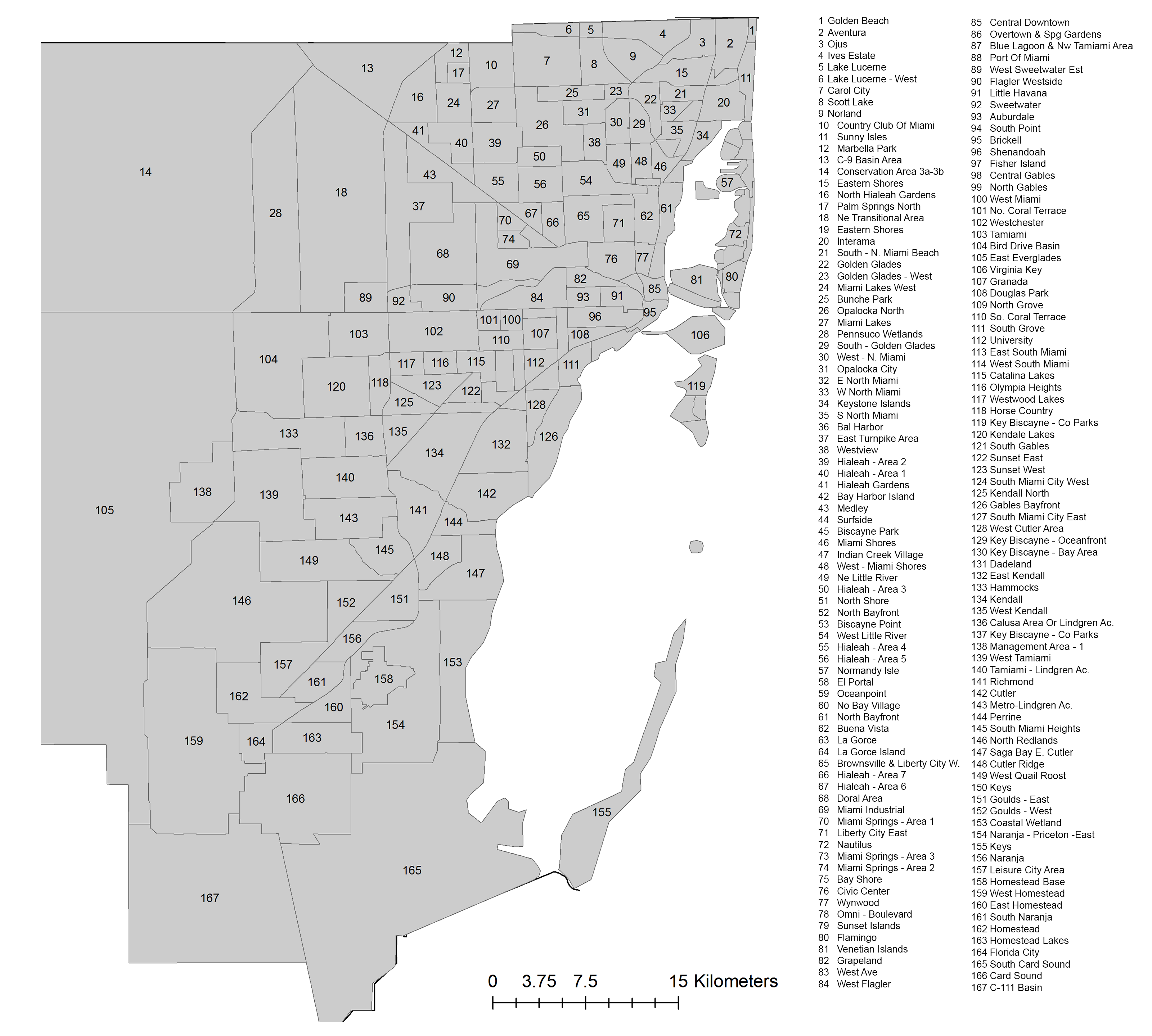

Supplement: Supplementary file 2 — Supplementary Information [file 41598_2019_51787_MOESM2_ESM.tif]
