# Supplementary material for: Urbanization creates diverse aquatic habitats for immature mosquitoes in urban areas
Source: Sci Rep. 2019 Oct 25;9:15335. doi: 10.1038/s41598-019-51787-5 (PMC6814835; doi:10.1038/s41598-019-51787-5)
Supplement: Supplementary file 3 — Supplementary Information [file 41598_2019_51787_MOESM3_ESM.docx]

**Supplementary table 1.** Most common breeding habitats.

| **Breeding Habitat** | **Number of Inspections** |
| --- | --- |
| Bromeliad | 598 |
| Storm Drain | 346 |
| Bucket | 247 |
| Flower Pot | 158 |
| Fountain | 134 |
| Tire | 116 |
| Plastic Container | 100 |
| Planter | 64 |
| Tire | 44 |
| Plant | 41 |
