# Supplementary material for: Urbanization creates diverse aquatic habitats for immature mosquitoes in urban areas
Source: Sci Rep. 2019 Oct 25;9:15335. doi: 10.1038/s41598-019-51787-5 (PMC6814835; doi:10.1038/s41598-019-51787-5)
Supplement: Supplementary file 4 — Supplementary Information [file 41598_2019_51787_MOESM4_ESM.docx]

**Supplementary Table 2. Mosquito aquatic habitats and respective categories.**

| Category 1 | Category 2 | |
| --- | --- | --- |
| **All Habitats** | **Artificial** | **Natural** |
| Baby Bath | Baby Bath | Bromeliads |
| Bag Of Chips | Bag Of Chips | Hole In Ground |
| Base | Base | Palm Leaf |
| Basketball | Basketball | Plants |
| Bird Bath | Bird Bath | Ponds |
| Bird Cage | Bird Cage | Puddle |
| Boat | Boat | Rock Hole |
| Bowl | Bowl | Tree Hole |
| Brick | Brick |  |
| Bromeliads | Bucket |  |
| Bucket | Can Of Paint |  |
| Can Of Paint | Christmas Tree Holder |  |
| Christmas Tree Holder | Concrete Floor |  |
| Concrete Floor | Construction Site |  |
| Construction Site | Container |  |
| Container | Containment Pallet |  |
| Containment Pallet | Cooler |  |
| Cooler | Cover |  |
| Cover | Cup |  |
| Cup | Dirt Spreader |  |
| Dirt Spreader | Dishwashing Machine |  |
| Dishwashing Machine | Doll House |  |
| Doll House | Drain |  |
| Drain | Dump |  |
| Dump | Dumpster |  |
| Dumpster | Feeder |  |
| Feeder | Fish Tank |  |
| Fish Tank | Flower Pot |  |
| Flower Pot | Fountain |  |
| Fountain | Garbage Can |  |
| Garbage Can | Garden Rake |  |
| Garden Rake | Holes On Floor |  |
| Hole In Ground | Jacuzzi |  |
| Jacuzzi | Jet Ski |  |
| Jet Ski | Ladder Steps |  |
| Ladder Steps | Metal Cover Pool |  |
| Metal Cover Pool | Nylon Cover |  |
| Nylon Cover | Planter |  |
| Palm Leaf | Plastic Bag |  |
| Plants | Plastic Bins |  |
| Planter | Plastic Boat |  |
| Plastic Bag | Plate |  |
| Plastic Bins | Pool |  |
| Plastic Boat | Pool Fence Holes |  |
| Plate | Pool Pump |  |
| Ponds | Pool Toy |  |
| Pool | Pot |  |
| Pool Fence Holes | Pot Plate |  |
| Pool Pump | PVC Pipe |  |
| Pool Toy | Roof |  |
| Pot | Sandbox |  |
| Pot Plate | Sink |  |
| Puddle | Slide |  |
| PVC Pipe | Storm Drain |  |
| Rock Hole | Stroller |  |
| Roof | Styrofoam Cup |  |
| Sandbox | Table |  |
| Sink | Tarp |  |
| Slide | Tire |  |
| Storm Drain | Toilet |  |
| Stroller | Toys |  |
| Styrofoam Cup | Tuna Can |  |
| Table | Uninstalled Sink |  |
| Tarp | Vase |  |
| Tire | Vehicle Port |  |
| Toilet | Washing Machine |  |
| Toys | Water Feeder |  |
| Tree Hole | Wheel |  |
| Tuna Can |  |  |
| Uninstalled Sink |  |  |
| Vase |  |  |
| Vehicle Port |  |  |
| Washing Machine |  |  |
| Water Feeder |  |  |
| Wheel |  |  |
|  |  |  |
